# Climatic influence on the growth pattern of *Panthasaurus maleriensis* from the Late Triassic of India deduced from paleohistology

**DOI:** 10.7717/peerj.9868

**Published:** 2020-09-08

**Authors:** Elżbieta M. Teschner, Sanjukta Chakravorti, Dhurjati P. Sengupta, Dorota Konietzko-Meier

**Affiliations:** 1 Institute of Biology, University of Opole, Opole, Poland; 2 Section Paleontology, Institute of Geosciences, University of Bonn, Bonn, Germany; 3 Geological Studies Unit, Indian Statistical Institute, Kolkata, West Bengal, India

**Keywords:** *Panthasaurus maleriensis*, Temnospondyli, Late triassic, Paleohistology, Histovariability, Climatic influence

## Abstract

Metoposaurids are representatives of the extinct amphibian clade Temnospondyli, found on almost every continent exclusively in the Late Triassic deposits. Osteohistologically, it is one of the best-known temnospondyl groups, analyzed with a wide spectrum of methods, such as morphology, morphometry, bone histology or computed modelling. The least known member of Metoposauridae is *Panthasaurus maleriensis* from the Pranhita-Godavari basin in Central India, being geographically the most southern record of this family. For the first time the bone histology of this taxon was studied with a focus on the intraspecific variability of the histological framework and the relationship between the observed growth pattern and climatic and/or environmental conditions. The studied material includes thin-sections of five long bones, a rib, an ilium and an intercentrum belonging most likely to eight individuals ranging from different ontogenetic stages. All bones have a large medullary region with progressively increasing remodeling, surrounded by a lamellar-zonal tissue type. The primary cortex consists of parallel-fibered matrix showing various degrees of organization, less organized collagen fibers in the zones and higher organized in the annuli. Growth marks occur in the form of alternating zones and annuli in every bone except the ilium and the intercentrum. The vascularity becomes less dense towards the outermost cortex in all sampled limb bones. Towards the outermost cortex the zone thickness is decreasing, in contrast to the avascular annuli, that become thicker or are of the same thickness. The growth pattern of *P. maleriensis* is uniform and represents changes in ontogenetic development. Multiple resting lines are prominent in the outer annuli of the limb bones and the rib and they presumably indicate climatic and environmental influence on the growth pattern. Therefore, a prolonged phase of slowed-down growth occurred during the unfavorable phase, but a complete cessation of growth indicated by Lines of Arrested Growth (LAGs) is not recorded in the studied samples. Based on the histological framework we conclude that the climate had an impact on the growth pattern. As we do not see any LAGs in the Indian metoposaurid, we assume that the local climate was relatively mild in India during the Late Triassic. A similar prolonged phase of slowed down growth without the occurrence of LAGs was observed in *Metoposaurus krasiejowensis* from the Late Triassic of Krasiejów (Poland). This is in contrast to Moroccan metoposaurid *Dutuitosaurus ouazzoui* from the Late Triassic of Argana Basin, where LAGs are regularly deposited throughout ontogeny indicating most likely harsher climatic conditions.

## Introduction

Bone histology is a powerful tool which allows scientists to study various, morphologically often not accessible, aspects of extinct animals’ biology. Through the microstructural framework and histological characters ontogeny, phylogeny, biomechanics, and environment could be revealed (Horner, de de Ricqlès & Padian, 1999; Horner, de de Ricqlès & Padian, 2000; de Ricqlès et al., 2001; Padian, de de Ricqlès & Horner, 2001; Padian & Lamm, 2013). The effects of the local environmental conditions are especially important for ectothermic animals e.g., amphibians, as for them the ambient environment has a direct influence on the formation of the bone tissue (Padian & Lamm, 2013). For ectothermic animals, the growth rate is directly related to environment. i.e., during favorable conditions, the bone deposition rate is usually higher, whereas during unfavorable conditions growth rate is relatively low or even cessation of growth could be observed (Francillon-Vieillot et al., 1990). Thus, based on the sequences of zones, annuli and lines of arrested growth (LAGs), a conclusion about the conditions in which the animal was living is possible. However, the histological bone framework is modified not only by the external factors, but is also biologically determined by ontogeny or genetic preconditions resulting in the process of developmental plasticity. For fossil taxa the direct determination which factors play more important role in creating the final bone structure and distinguishing between the influence of the internal and external factors on the development is extremally difficult, due to the lack of most of the biological information. The only possibility to determine the influence of the local conditions on the growth pattern are indirect methods based on the combination of the geological and histological information. To conduct such a study, a taxon with a wide geographical distribution, recognized geological setting and a well-known histological record is necessary. An ideal model for such test is the temnospondyl amphibian family occurring in the Late Triassic, namely Metoposauridae.

### Temnospondyli and Metoposauridae

Temnospondyli Zittel, 1888 is a large clade of extinct amphibians and their fossil remains can be found worldwide. It is represented by diverse groups with a notable variation in skull shape and body size. Their stratigraphical occurrence ranges from the Early Carboniferous (Holmes & Carroll, 1977) to the Early Cretaceous (Milner, 1990; Warren, Rich & Vickers-Rich, 1997). They occupied various ecological niches e.g., aquatic, semi-aquatic and terrestrial (Lindeman, 1991; Pawley & Warren, 2004; Schoch, 2014). The Metoposauridae is a group placed within the Stereospondyli clade (Schoch, 2014; Fortuny et al., 2018) with a stratigraphic appearance restricted to the Late Triassic (Schoch, 2003; Schoch, 2013). The group includes the European genera *Metoposaurus diagnosticus* (Von Meyer, 1842) (Germany), *Metoposaurus krasiejowensis* (Sulej, 2002) (Poland) and *Metoposaurus algarvensis* Brusatte et al., 2015 (Portugal), the African taxa from Morocco *Dutuitosaurus ouazzoui* (Dutuit, 1976), *Arganasaurus lyazidi* (Dutuit, 1976), *Arganasaurus azerouali* (Dutuit, 1976), and those from Madagascar *_Metoposaurus hoffmani* Dutuit, 1978, *nomen dubium* (Fortuny et al., 2019). Finally, in North America metoposaurids are represented by *Koskinonodon perfectus* (Case, 1922), *Anaschisma browni* (Branson, 1905) and *Apachesaurus gregorii* Hunt, 1993. From Asia, the Indian taxon *Panthasaurus maleriensis* (Roychowdhury, 1965) is known from the Late Triassic (Carnian to Norian) Maleri Formation (Roychowdhury, 1965; Sengupta, 2002; Chakravorti & Sengupta, 2019) based on multiple skeletal elements and from the Late Triassic (Carnian to Norian) Tiki Formation (Sengupta, 1992) based on only fragmentary preserved skull material. For a long time, *P. maleriensis* represented the most mysterious and least known taxon among all metoposaurids due to the unclear taxonomic relationship and its preservation state (Roychowdhury, 1965).

### Geology, environment and climatic conditions of the Maleri Formation

The Pranhita-Godavari Basin is one of the rift basins that were actively filled by the sediments when the Indian landmass was a part of the southern supercontinent Gondwana. The material used in this study originates from the Late Triassic Maleri Formation located within the Pranhita-Godavari Basin of Central India. The formation can be divided into lower and upper section (Kutty & Sengupta, 1988). However, no radiometric dating has been carried out so far. Biostratigraphically, the faunal assemblage of the Lower and Upper Maleri Formation is distinctive and non-overlapping (Datta, Ray & Bandyopadhyay, 2019). The Lower Maleri is considered Carnian in age based on the following index taxa viz. phytosaurs *Parasuchus hislopi* (Chatterjee, 1978) and *Volcanosuchus statisticae* Datta, Ray & Bandyopadhyay, 2019, the metoposaur *Panthasaurus maleriensis* (Roychowdhury, 1965), the rhynchosaur *Hyperodapedon huxleyi* (Mukherjee & Ray, 2014) a traversodontid *Exaeretodon statisticae* (Chatterjee, 1982) and a cynodont *Deccanodon maleriensis* (Nath & Yadagiri, 2007). The Upper Maleri fauna is assigned to the Norian age based on the occurrence of the chigutisaurs: *Compsocerops cosgriffi* and *Kuttycephalus triangularis* (Sengupta, 1995) more derived, phytosaurs cf. *Leptosuchus* (Novas et al., 2010), sauropodomorphs (Bandyopadhyay, 2011; Bandyopadhyay & Ray, 2020; Kutty et al., 2007 ) and the general disappearance of rhynchosaurs*,* metoposaurs as well as basal phytosaurs. Identical to other Gondwana basins, the Maleri Formation is dominated by a sandstone-mudstone alternation with occasional calcirudites. Dasgupta, Ghosh & Gierlowski-Kordesch (2017) recently suggested the presence of small, ephemeral and vegetated swamps or ponds along the flow path of the channels in both formation units. Sedimentological evidence of the deposition of parallel-laminate and cross-bedded sheet sandstone within the thick succession of mudstones proves a fluvial environment of the Maleri Formation (Robinson, 1970; Sarkar, 1988; Dasgupta, Ghosh & Gierlowski-Kordesch, 2017). The early Late Triassic climate of the supercontinent Pangea is said to be arid to semi-arid in the interior of Gondwana (Mueller, Krystyn & Kürschner, 2016). Robinson (1970) suggested a warm and humid climate of the Maleri Formation due to the presence of red mudstones, although the presence of red color in mudstones is not a sufficient indicator for the paleoenvironment. According to Smoot & Olsen (1988), red and massive mudstones can be deposited under dry to wet conditions and their variation can be only understood by the preservation of texture; however, according to Patranabish-Deb & Fukuoka (1998), it is more likely that the dissolution of ferric illite from the abundant Proterozoic rocks surrounding the Pranhita Godavari Basin contributed towards the red coloration of the mudstones in the Maleri Formation. Kutty (1971) pointed out that the presence of unioids at the junction of the Lower and Upper Maleri Formation represents a well-watered swamp-like environment indicating prevalent humid paleoclimate. Sarkar (1988) showed the presence of high smectite content (48–75%) through Differential Thermal Analysis (DTA) and X-ray analysis, which indicated some amount of rainfall. On the other hand, the sporadic presence of barite coupled with the occurrence of displacive and replacive calcite cements together with caliches indicate a warm to hot climate with low seasonal rainfall (Sarkar, 1988). Overall, according to Sarkar (1988), the predominance of smectite in the sediment, the poor floral content of the Maleri Formation, and the paucity of evaporites point to a low seasonal rainfall in a semi-arid environment. However, an array of aquatic fauna, including the metoposaurids, chigutisaurids and phytosaurs, present within the Maleri Formation does not support this. Most of these analyses were done on the Maleri Formation as a whole without distinguishing its bordering (upper and lower) parts. Detailed changes in the paleoenvironment of the Maleri Formation from Carnian to Norian is a work in progress. The excavated spiral and non-spiral coprolites produced by fishes and piscivorous animals yielded gymnosperm pollen, apiculate trilete spores produced by pteridophytes and sparse fungal and algal spores together with an admixture of wood shreds, amorphous vegetable matter and fungal remains (Vijaya, Prasad & Singh, 2009). Hence there is an indirect evidence of vegetation from coprolites as well as from the presence of herbivores such as the rhynchosaurs or dicynodonts (Nath & Yadagiri, 2007; Mukherjee & Ray, 2014; Bandyopadhyay & Ray, 2020). Moreover, according to the latest work by Dasgupta, Ghosh & Gierlowski-Kordesch (2017) the climate was warm with seasonal rainfall, which lead to the production of the large amount of mud aggregates and vertic soils and a semi-humid or semi-arid setting has been proposed with prominent seasonality for the Late Triassic Maleri Formation.

### General temnospondyl osteohistology

The most suitable bones for osteohistological studies from the postcranial material are the limb bones sectioned at the midshaft plane, since they contain the most complete bone growth record as those elements ossify early in ontogeny (Ricqlès, 1983; Francillon-Vieillot et al., 1990; Chinsamy, 1993; Erickson & Tumanova, 2000; Horner, Ricqlès & Padian, 2000; Sander, 2000). During the last two decades many osteohistological studies on various postcranial elements from various temnospondyl clades were published e.g., de Ricqlès (1979) and de Ricqlès (2001) described the general histological growth briefly concluding that temnospondyl bones preserve lamellar-zonal tissue; Damiani (2000) studied Triassic temnospondyl femora from Australia showing that they possess a large medullary region filled by trabeculae and remains of calcified cartilage, highly vascularized primary bone tissue with an alternation of thick zones built by parallel-fibered bone and thin, avascular annuli built by lamellar tissue, and the absence of LAGs; Witzmann (2009) studied the histological growth pattern of the dermal bones of various temnospondyl groups; Sanchez et al. (2010a) and Sanchez et al. (2010b) focused on bone histology of the small-sized *Apateon* from the Permian resembling a thin, almost avascular cortex consisting of lamellar bone and a large open medullary cavity, moreover, they indicated a strong paleoenvironmental and paleoecological influence reflected in its growth; Witzmann & Soler-Gijon (2010) analyzed osteoderms of various temnospondyl amphibians and *Bystrowiella* showing a metaplastic development in *Plagiosuchus*, and in *Gerrothorax* and dissorophids periosteal ossification; Konietzko-Meier & Schmitt (2013) studied a Middle Triassic *Plagiosuchus* femur and observed a small medullary cavity, surrounded by endosteal bone, a thick but porous cortex and an incipient fibro-lamellar bone gradually passing into parallel-fibered bone, and towards the outermost cortex more lamellar bone and five LAGs; Konietzko-Meier, Shelton & Sander (2016) studied North American stegocephalians including *Eryops, Archeria* and *Diadectes* from the Briar Creek bonebed, which revealed a different growth pattern resembling five different histotypes explained by interspecific or intraspecific variability. It seems that the clade Metoposauridae is the best studied due to its preservation and a generous number of various skeletal elements is available for histological studies. Femora of the metoposaurid *Dutuitosaurus ouazzoui* have been studied by Steyer et al. (2004), observing fast initial growth rate concluded on the basis of wide and densely vascularized zones in juvenile individuals and a decrease of growth rate in adult specimens indicated by a decrease in vascular density and thickness of zones. The sexual maturity was estimated for the seventh year of life (Steyer et al., 2004) and the growth pattern was linked with the local seasonal environment changes. The osteohistological growth of almost every skeletal element of *Metoposaurus krasiejowensis* has been studied, e.g., limb bones (Konietzko-Meier & Klein, 2013; Konietzko-Meier & Sander, 2013; Teschner, Sander & Konietzko-Meier, 2018). In general, the growth of *Metoposaurus krasiejowensis* consists of primary cortex built up of parallel-fibered bone, and sometimes even incipient fibro-lamellar bone (Konietzko-Meier & Sander, 2013), which might be exclusively deposited in juvenile individuals (Konietzko-Meier & Sander, 2013). There is also a rich data set published on dermal bones in order to infer information about feeding ecology based on biomechanical reconstruction of metoposaurid skull (Gruntmejer, Konietzko-Meier & Bodzioch, 2016; Gruntmejer et al., 2019a; Gruntmejer et al., 2019b; Konietzko-Meier et al., 2018). Analyzes of vertebrae helped to establish histological ontogenetic stages, a method which allows a relative determination of individual age, based on various histological and microstructural characters of vertebrae, when the classical growth cycles are not developed (Konietzko-Meier, Bodzioch & Sander, 2012) and showed the variability between different temnospondyl groups which may be taxonomically important (Konietzko-Meier, Danto & Gądek, 2014). Various groups studied in Konietzko-Meier, Danto & Gądek (2014) seem to have an individual histological framework, however in Stereospondyli the calcified cartilage is preserved a long time in all ontogenetic stages, contrary to Dvinosauria and Eryopoidae which ossify early in ontogeny. The preliminary analysis on ribs (Gądek, 2012) showed a very avascular primary tissue. Gee, Parker & Marsh (2017) studied vertebrae of the North American metoposaurids *Koskinonodon perfectus* and *Apachesaurus gregorii* confirming a uniform growth among all metoposaurid intercentra.

Studies including osteohistological research on Indian temnospondyls are rare (Ray, Mukherjee & Bandyopadhyay, 2009; Mukherjee, Ray & Sengupta, 2010; Mukherjee, Sengupta & Rakshit, 2020). Ray, Mukherjee & Bandyopadhyay (2009) provided preliminary results on the osteohistology of limb bones, ribs, and intercentra of three temnospondyl taxa from different Indian Triassic localities. They studied a trematosaurid from the Early Triassic (Panchet Formation, Damodar Basin), a paracyclotosaurid from the Middle Triassic (Denwa Formation, Satpura Basin), and a chigutisaurid from the Late Triassic (Maleri Formation, Pranhita-Godavari Basin). The conclusion from Ray, Mukherjee & Bandyopadhyay (2009) paper was that the examined taxa show distinct growth patterns: the trematosaurid exposed a thick layer of avascular lamellar bone at the periphery, towards the inner cortex a fibro-lamellar bone tissue, the absence of annuli or LAGs except for one humerus, and a woven fibered bone matrix in the ribs; the paracyclotosaurid were characterized by lamellar bone, high vascularity in the humerus and low vascularity in the femur and tibia, the ribs exhibited woven fibered bone matrix, and annuli and LAG were absent except for three LAGs in an intercentrum; the chigutisaurid shows a predominantly lamellar tissue in the long bones, woven fibered matrix in the rib, absence of growth rings in rib and intercentrum but presence of three LAGs in the humerus. The general conclusion was that the Early Triassic trematosaurids had an overall fast growth, in contrast to that of the Middle and Late Triassic temnospondyls (Ray, Mukherjee & Bandyopadhyay, 2009). Mukherjee, Ray & Sengupta (2010) restudied the material used in Ray, Mukherjee & Bandyopadhyay (2009), and they described the histology of bones from the families Trematosauridae (Early Triassic; humerus and femur), Paracyclotosauridae (Middle Triassic; humerus, femur, tibia, rib and intercentra), Chigutisauridae (Late Triassic; humerus, rib and intercentrum) and an indeterminate temnospondyl (Early Triassic; humerus and ribs) from India. In general, all samples showed a relatively compact cortex surrounding a well-differentiated central medullary region, the occurrence of predominantly longitudinal canals, a decreasing vascularity towards the outer cortex until an avascular outer cortex and the absence of growth rings except for three samples. In conclusion, a change of growth, during the ontogeny, from well-vascularized fibro-lamellar bone to peripheral lamellar bone and LAGs was observed in the Early Triassic trematosaurid, in contrast to the Middle Triassic paracyclotosaurid and Late Triassic chigutisaurid (predominance of lamellar bone), where the growth was cyclical but slow. In conclusion, the rapid growth in trematosaurids was explained as an adaptation to the free niche after the Permo-Triassic extinction event. Based on the growth pattern, the low to moderate cortical porosity in the Early Triassic taxon was linked with a terrestrial lifestyle, and due to a high cortical porosity and extensive medullary spongiosa observed in the analyzed chigutisaurid, a semi-aquatic to aquatic life mode was assumed. The varying cortical thickness in the paracyclotosaurid was explained with different biomechanical adaptation. No conclusions on the influence from the climate were drawn in that study.

In the study by Mukherjee, Sengupta & Rakshit (2020), a larger dataset was analyzed histologically including 17 limb bones ranging from juvenile to adult ontogenetic stages. The studied taxa included Middle Triassic capitosaurids, such as *Cherninia denwai* (nine limb bones), *Paracyclotosaurus crookshanki* (five limb bones) and an indetermined capitosaurid (five limb bones). The analyzes showed differences in paleobiology and lifestyle adaptations. The deposition of incipient fibro-lamellar bone tissue in temnospondyls was linked with the rapid achievement of a large body sizes. *C. denwai* shows first highly vascularized woven fibered bone tissue and later in ontogeny incipient fibrolamellar bone tissue with visible growth marks. The change in tissue type was linked to the achievement of the sexual maturity. The limb bones of *P. crookshanki* consist of parallel-fibered bone and azonal lamellar bone tissue during the entire ontogeny, implying a slow growth. For the life habitat reconstruction *C. denwai* is proposed to be a passive predator living on the bottom of the water reservoir, whereas *P. crookshanki* was reconstructed as a shallow water predator with certain level of terrestriality.

However, a clear correlation between osteohistology and the climatic influence was pointed out only in few papers. Sanchez & Schoch (2013) combined paleoecological and paleohistological analyses on the Triassic taxon *Gerrothorax* from two German localities and concluded an ecological flexibility and evidence of developmental and metabolic plasticity in the taxon. Konietzko-Meier & Klein (2013) and Konietzko-Meier & Sander (2013) observed an unusual alternation of fast (zones) and slow (annuli containing multiple resting lines) growth phases of almost the same thickness in *Metoposaurus krasiejowensis*, corresponding with the favorable and unfavorable seasons. Moreover, Konietzko-Meier & Klein (2013) compared the histological pattern known from femora of *M. krasiejowensis* with the results published for *Dutuitosaurus ouazzoui* in Steyer et al. (2004). The most characteristic difference is the structure of annuli and presence of typical LAGs in the African specimen. According to Konietzko-Meier & Klein (2013), this results from the occurrence of different local conditions, the climate in Morocco was harsh with distinct dry seasons resulting in a clear cessation of growth creating LAGs. In contrast, the mild climate in Krasiejów allowed the animals to grow almost the entire time and only a little influence of the dry period is observable as a decrease in growth rate. McHugh (2014) studied the Late Permian stereospondyl *Rhinesuchus* which shows a seasonal growth with a moderate remodeling, fibro-lamellar and lamellar bone tissue and zones and annuli of a various thickness. Moreover, the preservation of multiple resting lines in the annuli indicates slower metabolism during harsh climatic periods and a possible reason for Stereospondyli to overcome the Permo-Triassic extinction. McHugh (2015) analyzed humeri of *Micropholis stowi* (Dissorophoidea) and *Lydekkerina huxleyi* (Lydekkerinidae) from South Africa showing a convergent occurrence of fibro-lamellar tissue and the absence of LAGs and a suggestion of a terrestrial lifestyle for *M. stowi* due to a free medullary cavity, an azonal tissue, suggesting an adaptation to the dry and hot environmental conditions during the Early Triassic. Canoville & Chinsamy (2015) reported on the histological growth of *Lydekkerina huxleyi* concluding an overall faster growth in early ontogeny linked with a faster attended sexual maturity, moreover, the limb bone microanatomy and histology reveals an empty medullary cavity and a thick cortex implying an amphibious lifestyle with a tendency to more terrestrial or occasionally fossorial lifestyle probably as an adaptation to the harsh environmental conditions.

### Aim of the study

This study focuses on the change of the histological growth pattern within one species, namely *Panthasaurus maleriensis* originating from the Lower Maleri Formation in India. It is, so far, the only known metoposaurid from the southern hemisphere. The osteohistological growth of *P. maleriensis* has not yet been described and therefore the growth development within different skeletal elements in different ontogenetic stages of a single taxon is studied here. The Indian taxon is interesting for histological studies, as it was paleogeographically separated from other metoposaurid taxa and the climatic conditions based on geological studies are well-known (Dasgupta, Ghosh & Gierlowski-Kordesch, 2017). It allows us to test to what degree the osteohistology among metoposaurids is plastic and reflects the environmental condition. The comparison of the histological framework of three taxa, namely *P. maleriensis*, *M. krasiejowensis* and *D. ouazzoui* can help to answer the question about limits of developmental plasticity of bone structure among metoposaurids.

## Material & Methods

### Material

The sampled material belongs to the Indian representative of metoposaurids *Panthasaurus maleriensis* (Roychowdhury, 1965). The material was excavated near the Aigerapalli village in the Lower Maleri Formation from the Late Triassic (Sengupta, 1995; Sengupta, 2002; Sengupta, 2003; Chakravorti & Sengupta, 2019). Morphologically, the cranial and postcranial material of *Panthasaurus maleriensis* were described in detail first in Roychowdhury (1965) and later in Sengupta (2002). The here analyzed samples are restricted mostly to limb bones including two humeri, one femur, a tibia and a fragmentary ulna. In addition, a rib, an ilium, and an intercentrum of *P. maleriensis* were sectioned. Both humeri studied here are from the left body size, where the smaller humerus (ISIA 73) measures 65 mm in length with a diameter of 24 mm, and the larger humerus (ISIA 70) measures 120 mm in length, and 35 mm in midshaft diameter. The femur (ISIA 83) is from a left limb and has a total length of 140 mm and a midshaft width of 26 mm. The tibia (ISIA 98) has a length of 62 mm and the fragmentary preserved (posterior portion) ulna (ISIA 200) measured 32 mm in length. The reconstructed approximate length of the entire bone is 64 mm. In addition, we studied a left ilium (ISIA 87) measuring 51 mm in length and a fragmentary rib (ISIA 199) with a preserved length of 37 mm. No proxy is available for a total length reconstruction of the rib. The intercentrum (ISIA 198) has a height of 41 mm and a width of 40 mm. The material is stored in the collection of the Indian Statistical Institute (ISI) in Kolkata. All studied bones are listed in Table 1. The maximal known size of a *P. maleriensis* femur is 140 mm (ISIA 83), of a humerus is 144 mm (ISIA 68), of an ulna is 78 mm (ISIA 97) and of a tibia is 65 mm (ISIA 99).

**Table 1:** Measurements taken from the the sectioned bones of *Panthasaurus maleriensis*.

**Skeletal element of *Panthasaurus maleriensis***	**Specimen number**	**Total length**	**Percentage of largest bone**	**Midshaft width**	**Visible cycles**	**Estimated cycles**
Humerus (L)	ISIA 73	65	45%	24	1,5	1,5
Humerus (L)	ISIA 70	120	83%	35	3	4
Femur (L)	ISIA 83	140	100%	26	4	5
Ulna (R) fragm.	ISIA 200	64^a^	82%^a^	11	2	3
Tibia (R)	ISIA 98	62	95%	12	3	4
Rib fragm.	ISIA 199	37	–	14	3	–
Ilium (L)	ISIA 87	51	56%	13	–	–
Intercentrum	ISIA 198	41	–	43	–	–

**Notes.**

L left R right fragm. fragmentary

a estimated length.

All taken measurements of Panthasaurus maleriensis bones are in millimeters.

### Methods

#### Thin-sectioning

The thin-sections have been prepared in the laboratory of the Institute of Geosciences at the Rheinische Friedrich-Wilhelms-Universität Bonn, Germany. All bones were sectioned according to Stein & Sander (2009) and Lamm (2013), however, their technique was slightly modified. Wet silicon carbide (SiC) grinding powder with grit sizes of 600 and 800 were used for grinding and polishing the thin-sections. All limb bone sections were cut at the midshaft plane. A sagittal section of the intercentrum was made. The osteohistological analysis was performed with a LEICA DM LP polarizing light microscope and the photographs were taken with a LEICA DFC 420 camera attached to the microscope. The sections were scanned with an EPSON PERFECTION 750V PRO scanner in order to gain microstructural overview.

#### Measurements of the growth cycles thickness

For the estimation of the width of the growth cycles (= zone + ) we calculated separately the relative percentage thickness for the zones and annuli in correlation to the total cortex width (Table 2).

#### Terminology

The morphological description follows the nomenclature used in Sulej (2007), where the osteohistological nomenclature is based on Francillon-Vieillot et al. (1990). Here, we distinguish between two types of Sharpey’s fibers, first representing long fibers reaching deep into the cortex, being most probable muscle and tendon attachments (Francillon-Vieillot et al., 1990) and second, short fibers arranged in bundles, existing only close to the periosteal surface, being probably attachments of the periosteum (Witzmann, 2009; Konietzko-Meier & Sander, 2013). The meaning of zone and annulus in the current study follows Konietzko-Meier & Sander (2013). The zone is a highly vascularized layer, with lower organization of collagen fibers in contrast to the term annulus, which refers to the layer without or low number of vascular canals and higher organization of collagen fibers. In the studied material no annual LAGs occur, instead, adjacent to the annuli, numerous lines are present, which are referred to in this paper as resting lines (for details see Discussion section and paper by Konietzko-Meier & Sander, 2013). The count of the annual growth cycles (= age in years) is based on a sequence of a zone and an annulus.

**Table 2:** Percentage thickness of the individual annuli and zones of Panthasaurus maleriensis limb bones.

*Panthasaurus maleriensis* skeletal element vs. growth cycle	Humerus ISIA 73		Humerus ISIA 70		Femur ISIA 83		Ulna ISIA 200		Tibia ISIA 98	
	zo	an	zo	an	zo	an	zo	an	zo	an
1st cycle	40	10	-^*^	-^*^	-^*^	-^*^	-^*^	-^*^	-^*^	-^*^
2nd cycle	50		53	13	35	5	59	4	54	19
3rd cycle			6	**14**	7	**7**	8	**29**	5	**12**
4th cycle			6	**8**	7	**18**			5	**5**
5th cycle					7	**14**				

**Notes.**

zo zone an annulus

* estimated length

All values are calculated in % as a proportion of the individual width of every zone and annulus versus the total cortex width; bold are marked all ratios where the annulus is thicker than the zone in one growth cycle.

## Results

### General osteohistology of *Panthasaurus maleriensis*

**Figure 1:**
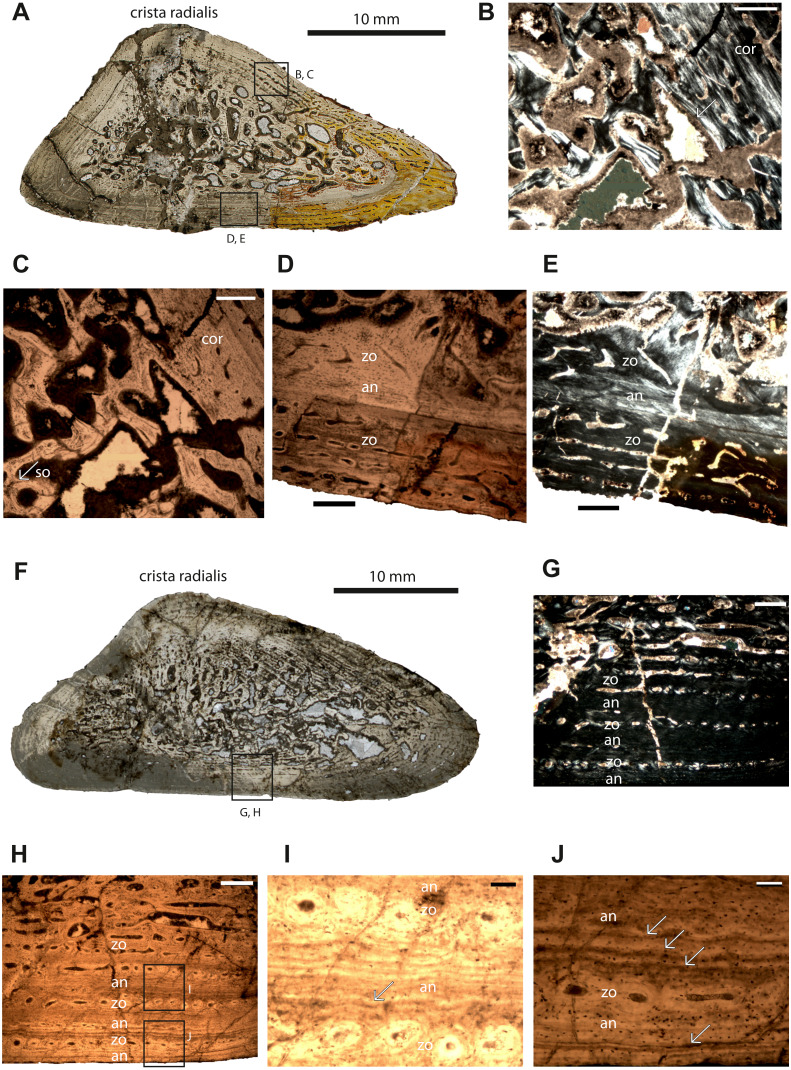
Microstructure and histology of the humeri of *Panthasaurus maleriensis*. (A) microstructure of the small-sized humerus ISA 73. (B) the unsharp border between the cortex (cor) and the perimedullary cavity, the secondary osteon (so) is indicated by an arrow; in normal transmitted light. (C) the same picture as in (B) but in polarized light. (D) border between the innermost cortex, a thin annulus and the outermost cortex; a change of vascularization from reticular to longitudinal towards the periosteal surface can be observed; in normal transmitted light. (E) the same picture as in (D) but in polarized light. (F) microstructure of the large-sized humerus ISA 70. (G) section showing the alternation of zones and annuli; perimedullary region showing large erosion cavities; towards the cortical surface the vascular canals show a change in shape, from longitudinal to reticular; the width of the annuli increases towards the sections surface; in normal transmitted light. (H) the same picture as in (G) but in polarized light. (I) enlargement of (H) showing two zones consisting of single vascular rows and an annulus with multiple resting lines; in normal transmitted light. (J) enlargement of (H) showing a wide annulus, a zone with a single row of vascular canals and the outermost annulus; the multiple resting lines are indicated by arrows; in normal transmitted light. Scale bar: (B, C, D, G), 500 µm; (I and J), 100 µm. Abbreviations: cor, cortex; so, secondary osteon; zo, zone; an, annulus.

The innermost part of every sectioned bone (Table 1) is built up by secondary trabecular bone, which is followed by a perimedullary region consisting of a mix of primary and secondary bone. The border between the medullary region, perimedullary region and the cortex is not well defined (Fig. 1A). The primary cortex is preserved throughout the complete section in every specimen, although the bone remodeling is in different stages. The primary matrix consists of parallel-fibered bone with various degrees of collagen-fibers organization (Figs. 1B–1C). Vascularization varies from secondary osteons and/or resorption cavities visible in the deep layers of the cortex (Figs. 1B–1C) and towards the periosteal margin it is present as simple vascular canals and/or primary osteons in the outermost part of the cortex. The orientation of the vascular canals varies from longitudinal and reticular in the innermost cortex to longitudinal canals arranged in rows towards the outermost cortex (Figs. 1D–1J) in all samples. Usually, only zones (with lower organized collagen fibers and numerous vascular canals) and annuli (with higher organized collagen fibers and limited amount of vascular canals) occur as growth marks, and a complex build of a zone and an annulus is counted here as one annual growth cycle (Figs. 1G–1H). No classical LAGs are observed. Osteocyte lacunae are numerous in every section.

### Detailed osteohistologicial description

**Humeri** –Humerus ISIA 73 represents a small-sized humerus consisting of 45% of the maximal length of the largest known humerus (ISIA 68). The cross-section is triangular in shape (Fig. 1A). The medullary region is filled with secondary trabeculae (Figs. 1B–1C). The perimedullary region is rather thin in comparison to the cortical thickness and still preserves a relatively large amount of primary tissue. The erosion cavities are larger on the anterior side. The bone matrix is dominated by highly organized parallel-fibered tissue. In the innermost part of the section vascular canals are longitudinal to reticular. Towards the outer cortex a change of simple vascular canals shape is visible (Figs. 1D–1E); the canals are longitudinal. On the ventral side, the canals become radial in shape. Primary osteons dominate in this specimen. A sequence built of the first, innermost zone and an annulus is followed by a second, outermost zone (Figs. 1D–1E, Table 2). Generally, the zones are wider than the annulus in this specimen. The first visible zone has almost been destroyed by remodeling (40% of the cortex, Table 2). The following first avascular annulus is thin (10% of the cortex, Table 2). The second visible zone is thicker than the first zone (50% of the cortex, Table 2) and it possesses rows of longitudinal and reticular vascular canals. No LAGs or resting lines are observed in this section. Short Sharpey’s fibers are present, and they occur close to the sections’ surface, mostly on the posterior and anterior tip of the bone, although they are not very abundant.

Humerus ISIA 70 represents a large-sized humerus consisting of 83% of the maximal length (ISIA 68). The cross-section is triangular in shape (Fig. 1F) and the nutrient canal is visible on the ventral side. The resorption is extensive and the perimedullary region is well developed (Fig. 1F). Deep in the primary cortex, a mix of longitudinal and reticular vascular canals is preserved, and towards the outer cortex the canals become more longitudinal, are sometimes connected to each other, and are arranged in rows (Figs. 1G–1H). In the region of the *crista radialis*, the vascular canals are mostly reticular in shape. In this specimen the zones and annuli vary in thickness (Table 2). Three growth cycles can be distinguished, and after the first cycle a change of growth rate is visible (Figs. 1G–1H). The innermost first visible zone is wide (53% of the cortex, Table 2) and highly vascular but contains also numerous erosion cavities. Following this the first visible, thin annulus (13% of the cortex, Table 2) with a few resting lines (Fig. 1I) is laid down and the second zone comprising of only one row of longitudinal vascular canals (6% of the cortex, Table 2) is deposited. It is followed by a thick (13% of the preserved cortex, Table 2) second annulus (Figs. 1G–1J), which shows multiple resting lines (Fig. 1J). Towards the subperiosteal surface the third, thin zone containing only one row of vascular canals occurs (6% of the cortex, Table 2). The third annulus is again thick (8% of the cortex, Table 2) and it possesses multiple resting lines (Fig. 1J). The Sharpey’s fibers are not very prominent in this section, although they occur close to the cortical surface on the dorsal side.

**Figure 2:**
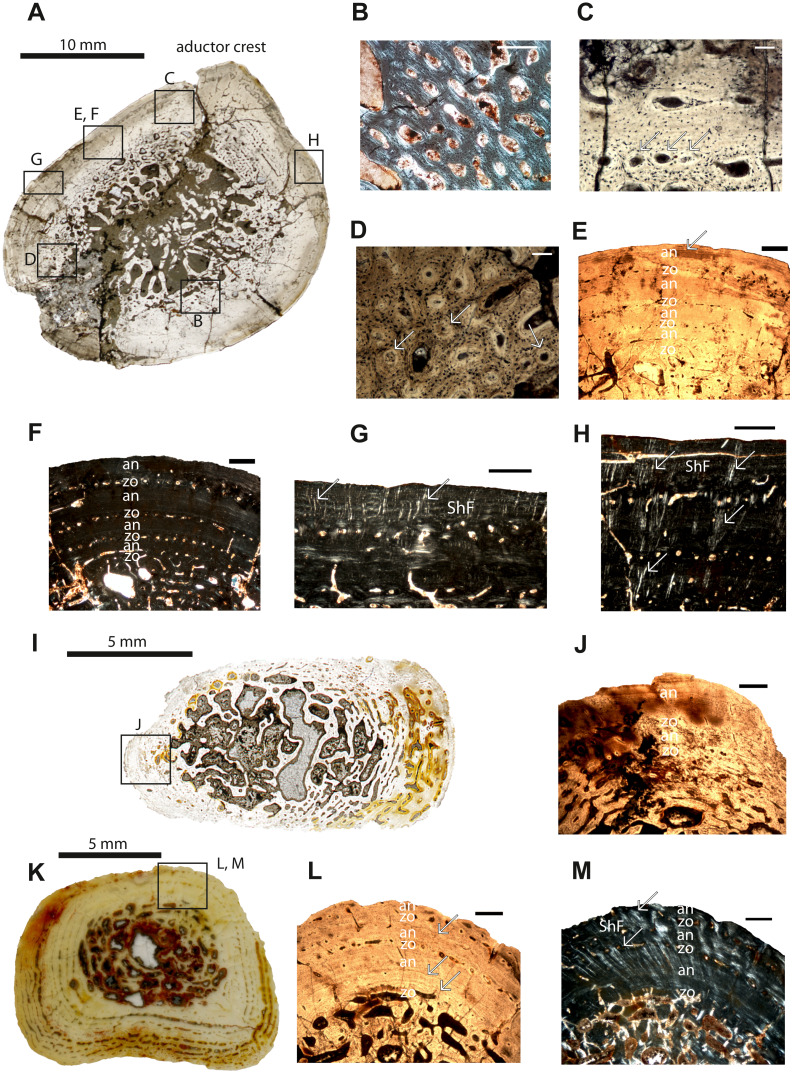
Microstructure and histology of the femur, ulna and tibia of *Panthasaurus maleriensis*. (A) microstructure of the femur ISA 83. (B) incipient fibro-lamellar tissue; in polarized light. (C) primary osteons embedded in the bone matrix indicated by arrows; in normal transmitted light. (D) secondary osteons embedded in the bone matrix, with a visible cementum line indicated by arrows; in normal transmitted light. (E) section showing the alternation of zones and annuli; perimedullary region showing erosion cavities; towards the sections surface the zones decrease and annuli increase in width; towards the surface the vascular canals become more longitudinal with an arrangement in single rows; in normal transmitted light. (F) the same as in (E) but in polarized light. (G) short Sharpey’s fibers indicated by arrows, occurring close to the cortical surface with an arrangement in bundles; in polarized light. (H) long Sharpey’s fibers indicated by arrows reaching inside the cortex; in polarized light. (I) microstructure of the ulna ISA 200. (J) section showing an alternation of zones and annuli; in normal transmitted light. (K) microstructure of the tibia ISA 98. (L) section showing the alternation of zones and annuli; perimedullary region showing large erosion cavities; the zones are thin with only one row of vascular canals; the annuli are much thicker than the zones; arrow indicates a resting line in an annulus close to the sections surface; in normal transmitted light. (M) the same as in (L) but in polarized light; the arrows indicate Sharpey’s fibers which are long and reach up into the cortex. Scale bar: (B, C, D), 100 µm and (E, F, G, H, J, L and M), 500; µm. Abbreviations: zo, zone; an, annulus; ShF, Sharpey’s fibers.

**Femur** –The cross-section of specimen ISIA 83 belongs to the largest preserved femur in the ISI collection. The section is oval in shape (Fig. 2A). The medullary region is large, with a few secondary trabeculae preserved inside (Figs. 2A–2F). The perimedullary region is distinct and possesses many erosion cavities due to progressed process of remodeling. The vascular canals are mostly longitudinal (Fig. 2B) and the number of canals decreases towards the cortical surface. The primary osteons (Fig. 2C) are dominant in this section and they are arranged in rows. The section preserved also secondary osteons (Fig. 2D). Four annual growth cycles were observed (Table 2). After the appearance of a thick, first zone (35% of the cortex, Table 2), the thickness of the following zones decreases distinctly, whereas the annuli increase their thickness compared to the associated zones and become even distinctly thicker (Figs. 2E–2F; Table 2). In the first visible innermost zone (7% of the cortex, Table 2) the vascularization is very high and the organization level of the tissue is very low. The structure of tissue resembles the incipient fibro-lamellar bone (Fig. 2B). The following first visible annulus is very thin and avascular (5% of the cortex, Table 2). The second visible zone with one row of vascular canals (7% of the cortex, Table 2) can be distinguished followed by a second annulus (7% of the cortex, Table 2). The third visible zone has one row of vascular canals (7% of the cortex, Table 2) and then the third visible, thick annulus (18% of the cortex, Table 2) is laid down. The fourth visible zone (7% of the cortex, Table 2) consists of one row of vascular canals and the section ends with a comprised fourth annulus (14% of the cortex, Table 2). In all annuli numerous resting lines are visible, however, they are the most prominent in the third and fourth annulus. Sharpey’s fibers are very prominent in this section, preserving long fibers that extend deep into the cortex (on the anterior side) and a second type of short fibers that are arranged in bundles and are limited to the section’s outermost margin (Figs. 2G–2H).

**Ulna**–The cortex of the ulna sample (ISIA 200) is not completely preserved due to the fragmentary nature of the bone, however a fragment containing the outermost cortex is present (Figs. 2I–2J). A clear medullary region is not distinguishable, and the perimedullary region possesses large erosion cavities, since the remodeling process is highly advanced and has proceeded into the primary cortex. The vascular canals in the cortex are primarily longitudinal and few have become secondarily altered to reticular canals. Towards the outer cortex they become less numerous, but are arranged in rows. In general, two growth cycles are preserved (Fig. 2J). The innermost, first visible zone (59% of the cortex, Table 2) has been almost entirely remodeled and is bordered by a first visible annulus (4% of the cortex, Table 2), which is followed by the second visible, thin zone (8% of the cortex, Table 2) consisting of only one row of vascular canals. Next, a second visible, thick (29% of the cortex, Table 2) annulus with several prominent resting lines occurs. Sharpey’s fibers are not abundant in this section.

**Tibia**–The specimen ISIA 98 consists of 95% of the maximal length of the largest known tibia (ISIA 99). It has a small and open medullary cavity with visible remains of endosteal bone. The perimedullary region is large and the remodeling is extensive. The border to the primary cortex is sharp and clearly visible, and has a low degree of remodeling. In the cortex, the vascular canals are longitudinal and sometimes circumferential (Fig. 2K). Secondary osteons are dominant in the section. In general, three growth cycles are present (Figs. 2L–2M). The innermost, first visible zone is wide (54% of the cortex, Table 2), but scattered by large erosion cavities. However, the remains of the primary tissue indicate that it was once highly vascularized and low organized. Following this is a very thick, distinct annulus (19% of the cortex, Table 2) with numerous resting lines. Next, the second zone, consisting of only one row of vascular canals (5% of the cortex, Table 2), is followed by a thick annulus (12% of the cortex, Table 2), and lastly the third visible zone (5% of the cortex, Table 2) consisting again of only one row of vascular canals is deposited. The section ends with the third annulus (5% of the cortex, Table 2), which is incompletely altered (Figs. 2L–2M). All annuli possess many resting lines (Fig. 2L). Long and numerous Sharpey’s fibers are very prominent in this section (Fig. 2M).

**Figure 3:**
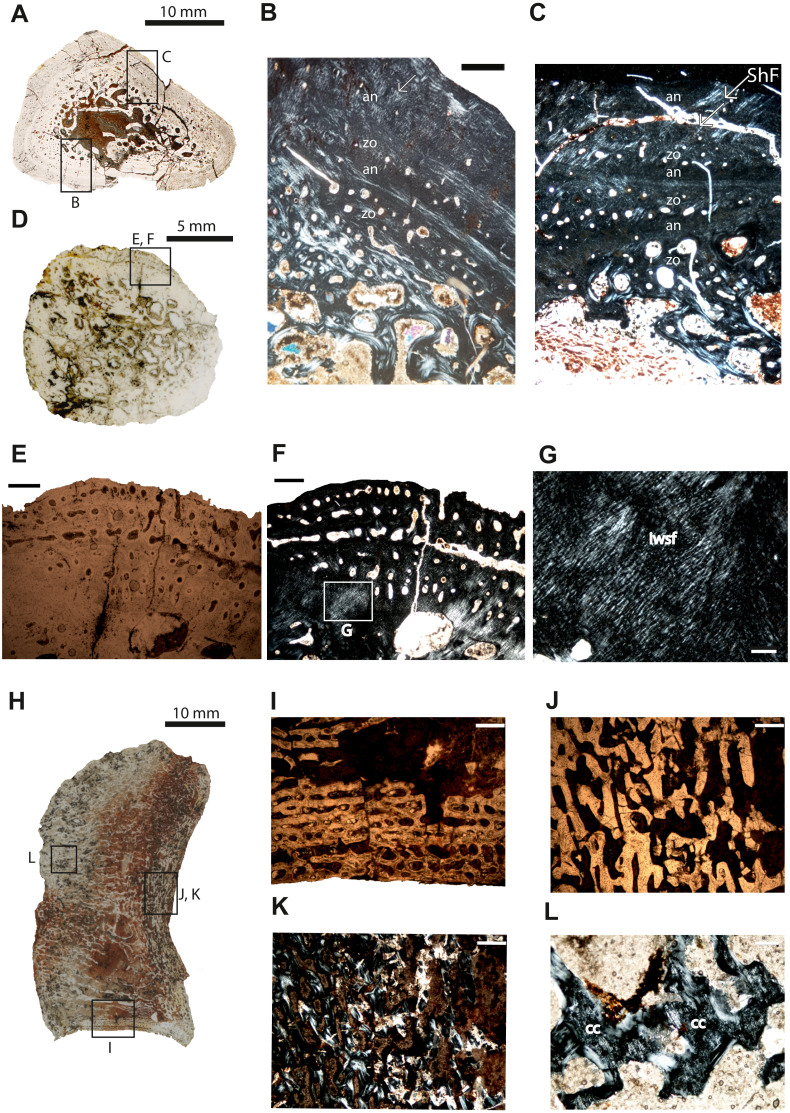
Microstructure and histology of the rib, ilium and intercentrum of *Panthasaurus maleriensis*. (A) microstructure of the rib ISA 199. (B) the section showing the alternation between zones and annuli; the vascularization is moderate; the zones in the bone’s center are thicker than towards the sections surface, in contrast to the annuli which increase in width towards the sections surface; in the outermost annulus metaplastic-like looking tissue occurs; these are interwoven structural fibers (iwsf). (C) the section ahows an alternation between zones and annuli and advanced remodeling; the arrow indicates Sharpey’s fibers which are long and reach up into the cortex. (D) microstructure of the ilium ISA 87. (E) the section showing no zones or annuli; the vascular canals are arranged on the posteromedial side; the remodeling process is advanced; in normal transmitted light. (F) the same as in (E) but in polarized light. (G) enlargement from (F) showing metaplastic-like tissue; in polarized light (H) microstructure of the intercentrum ISA 198. (I) primary trabecular bone with vascular canals arranged in rows in periosteal bone; in normal transmitted light. (J) secondary trabeculae visible in endochondral domain; in normal transmitted light. (K) the same as in (J) but in polarized light. (L) remains of calcified cartilage (cc); in polarized light. Scale bar: (B, C, E, F, I J, K), 500 µm; (G, L), 100 µm. Abbreviations: zo, zone; an, annulus; ShF, Sharpey’s fibers; iswf, interstractural woven fibers; cc, calcified cartilage.

**Rib**–The cross-section of specimen ISIA 199 is triangular in shape (Fig. 3A). The large medullary region consisting of secondary trabeculae shows a roughly central cavity (Figs. 3B–3C). Many primary osteons are observed within the bone matrix. The sample shows three preserved growth cycles (Figs. 3B–3C). In the first innermost zone rare but large erosion cavities appear, followed by a very thin annulus comprised of highly organized tissue. The second zone is thinner than the first zone and contains only few scattered longitudinal vascular canals. Next, a second annulus of approximately the same thickness as the first annulus has been laid down. Then the third zone is preserved with again only scattered vascular canals, with a similar thickness like in the second zone, however, with distinctly lower vascularization. The difference between the following annulus is visible in the lower organization of the parallel-fibered bone. The section finishes with an annulus which possesses very prominent resting lines. Long Sharpey’s fibers can be observed (Fig. 3C).

**Ilium**–The specimen ISIA 87 consists of 56% of the longest preserved individual. The cross-section from the midshaft is roundish in shape (Fig. 3D). The medullary region is extremely large surrounded by a perimedullary region showing extensive resorption (Figs. 3E–3F). The preserved cortex is very fibrous on the lateral side, reminiscent of a metaplastic-like bone with many structural interwoven fibers (Fig. 3G). Numerous vascular canals are limited to the outermost part of the cortex on the posteromedial and anteromedial sides (Figs. 3E–3F). No clear growth marks are visible in this section.

**Intercentrum**–Specimen ISIA 198 represents an intercentrum in sagittal-section (Fig. 3I). The bone histology is poorly preserved due to diagenesis. Periosteal bone is present only on the ventral side forming a triangular structure (Fig. 3H). The periosteal domain consists of parallel-fibered bone with rows of longitudinal vascular canals (Fig. 3I), however, the preservation state of the bone does not allow us to distinguish any more details. The largest part of this section is built up by bone tissue of endochondral origin (Figs. 3J–3K), but eroded and thus very poorly preserved. In the endochondral domain, irregular primary or secondary, trabecular bone is observed (Figs. 3J–3K). The primary trabecular bone is visible in the middle part of the section, which is poorly ossified with a large amount of calcified cartilage that is preserved between the trabeculae (Fig. 3L). Anteriorly and posteriorly the amount of secondary remodeled trabeculae increases. Neither growth marks nor Sharpey’s fibers are visible.

## Discussion

### Intra- and interskeletal variability in *Panthasaurus maleriensis*

To answer the questions how far the environment may change the genetically conditioned histological framework, it is necessary to distinguish which characters are biologically related and/or environmentally modeled. Thus, it is important to know how bones change during ontogeny, and how the environmental signal is coded. Ontogenetic or biomechanical changes are usually visible through changes of the histological characters along the growth series (age determination or histological ontogenetic stages, see: Klein & Sander, 2008) whereas the environmental signal is expressed by the growth pattern (Padian & Lamm, 2013).

All bones studied here show lamellar-zonal tissue, with clear alternation of zones and annuli. Annuli are built from highly organized parallel-fibered bone, whereas zones represent the low organization of the same matrix type. However, along the growth series the ontogenetic change of the growth marks is visible: the older the individual, the more the growth rate decreases and the more the tissue organization increases. Only in femur ISIA 83 (Fig. 2B) in the deep part of the cortex incipient fibro-lamellar bone has been observed, which suggests a fast-initial growth (Konietzko-Meier & Klein, 2013). The incipient fibro-lamellar bone would be most likely deposited for gaining size fast in order to overcome predation (Wiffen et al., 1995; Horner, Ricqlès & Padian, 2000; Steyer et al., 2004).

In all specimens with the preserved cyclical growth (except the ilium and the intercentrum) the innermost zone is thicker than the following annulus, and, toward the surface of the cortex, the zones become very thin and consist of only one row of vascular canals, whereas the outermost annuli are thicker and increase in width towards the outer cortex (Figs. 1D–1E, 1G–1J; 2E–2F, 2J, 2L; Table 2). Moreover, no clear LAGs have been observed, although the annuli show phases of prolonged slowed-down growth with several short-lasting periods of cessation of growth indicated by multiple resting lines.

Interesting is that in all bones, except the small humerus, starting with the third cycle and moving outwards, a significant change of the growth pattern is observed (Table 2) and annuli start to have the same thickness or become even thicker than zones. The progressive decreasing of growth rate would suggest an important event in the animal’s life cycle and could be related to reaching the point of sexual maturity (Castanet, 1975; Steyer et al., 2001; Steyer et al., 2004). Steyer et al. (2004) calculated the sexual maturity for *Dutuitosaurus* at the age of seven years based on the histological characters preserved in the femora (Table 3). For *P. maleriensis* the point of sexual maturity occurred earlier in the ontogeny and, based on the tested bones, should be calculated at the age of approximately three to four years (Table 3). For the taxon from Poland the age of achieving sexual maturity is not possible to determine, as all bones belong to juvenile individuals and signs of decreasing growth are not observed in the histological samples (Konietzko-Meier & Klein, 2013). Importantly, in none of the tested metoposaurids the age of achieving the maximal size is represented by a classical External Fundamental System (EFS) (de Ricqlès et al., 2000). In the two largest bones of *P. maleriensis* (femur ISIA 83 and humerus ISIA 70) in the outermost annulus, numerous and very distinct resting lines are visible (Figs. 1G–1J; 2E–2F, 2J) accompanied by the overall decreasing growth rate of the cortex. However, because similar lines occur also in deeper situated annuli it is not clear if the accumulation of the resting lines next to the cortical surface represents a structure similar to an EFS or if the well-visible resting lines are an effect of diagenetic processes. The same process of the preservation of multiple resting lines in the outer part of the cortex was observed for *M. krasiejowensis*, however in that case without a visible decreasing growth rate (Konietzko-Meier & Sander, 2013). Thus, we conclude that the decreasing growth rate observed in *P. maleriensis* is related to the adulthood although the animal did not achieve its maximal size.

**Table 3:** Number of visible and estimated cycles in different skeletal elements of *Panthasaurus maleriensis*, *Metoposaurus krasiejowensis* and *Dutuitosaurus ouazzoui*.

**Skeletal element**	**Taxon name**	**Total length [mm]**	**Visible cycles**	**Estimated cycles**	**Published as:**
Humerus	*Panthasaurus maleriensis*	65	1,5	1,5	Current study
Humerus	*Metoposaurus krasiejowensis*	67.1	1	1	Teschner, Sander & Konietzko-Meier (2018)
Humerus	*Panthasaurus maleriensis*	120	3	4	Current study
Humerus	*Metoposaurus krasiejowensis*	82.6	2	2	Teschner, Sander & Konietzko-Meier (2018)
Femur	*Panthasaurus maleriensis*	140	4	5	Current study
Femur	*Metoposaurus krasiejowensis*	92	2	3-4	Konietzko-Meier & Klein (2013)
Femur	*Dutuitosaurus ouazzoui*	148	3	10	Steyer et al. (2004)
Ulna fragment	*Panthasaurus maleriensis*	64^a^	2	3	Current study
Ulna	*Metoposaurus krasiejowensis*	36.9	2	2	Konietzko-Meier & Sander (2013)
Tibia	*Panthasaurus maleriensis*	62	3	4	Current study
Tibia	*Metoposaurus krasiejowensis*	55	4	–	Current study
Rib fragm.	*Panthasaurus maleriensis*	37	3	–	Current study

**Notes.**

a estimated length.

The remodeling process has begun in all samples but is the least-advanced in the ISIA 73 humerus, contrary to the larger humerus (ISIA 70) which has undergone the most extensive remodeling. In *P. maleriensis* we observe that the bones become more porous and therefore lighter (bone mass decrease) during ontogeny. A similar pattern is shown in *D. ouazzoui* bones, where the medullary region expands and the cortex becomes thinner during ontogeny (Steyer et al., 2004, Fig. 6). It could be connected to the change of the ecological niche, and the adaptation to a more aquatic lifestyle (Dutuit, 1967; Steyer et al., 2004; Gee & Parker, 2018). The histological framework supports the observation done by Dutuit (1976), who noted the taphonomic separation of adult (central basin) and juvenile (periphery of basin) specimens in the Argana Basin. This would indicate that while the animal is young and still fast growing, it would occupy a shallow water niche that would give it a safe place free of predation. As the animal matures, it might change the water column depth for obtaining food and overcoming predation on its own (Wiffen et al., 1995; Horner, de Ricqlès & Padian, 2000; Steyer et al., 2004). The same was concluded by Gee & Parker (2018), based on the absence of large-sized metoposaurids in the North American localities, which might be due to an ecological separation of juveniles and adult specimens. Also, in the Polish locality preserving *M. krasiejowensis*, a taphonomic preservation of juvenile individuals is dominant over the adult specimen, however in that case it is not a result of the biological segregation, but is connected with the mass segregation during transportation of the fossil material into the Lagerstätte (Bodzioch & Kowal-Linka, 2012).

Sharpey’s fibers occur in two different forms in both species, first type as bundles of short fibers (periosteum attachment) and second type as long fibers reaching deep into the cortex (muscle attachment). However, they are not abundant in *P. maleriensis* except in femur ISIA 83 and tibia ISIA 98. This does not correlate with the pattern in *M. krasiejowensis*, where numerous long and short Sharpey’s fibers are preserved in every skeletal element. Moreover, the preservation of numerous Sharpey’s fibers seems to be a typical character for temnospondyls observed also in North American *Eryops, Archeria* and *Diadectes* (Konietzko-Meier, Shelton & Sander, 2016), also visible in modern amphibians (Kolenda et al., 2018). Long Sharpey’s fibers being most likely attachments of muscles are very prominent in the femur and tibia sections (Figs. 2G–2H, 2M), in contrast to the humeri, where they are not abundant. There might be also a biological adaptation influencing the growth pattern, and therefore a probable different usage of hind limbs over front limbs linked most likely to locomotion (Carrier & Leon, 1990; Margerie de et al., 2004). It has been proposed for *M. krasiejowensis* that it might bury in the soft substrate to overcome an unfavorable period (Konietzko-Meier & Sander, 2013).

### Age estimation and interpretation of ontogenetic growth

A method in paleohistology which allows the retrocalculation of the lost or resorbed growth cycles to estimate the individual age of an animal is known as the superimposition method (Leclair Jr & Castanet, 1987; Castanet et al., 2004; Bybee, Lee & Lamm, 2006; Sanchez et al., 2010b). This method however, requires the application of a well-defined ontogenetic series, consisting of at least two bones from a different size range and, which in our case was only possible for the humeri. The other method to retrocalculate the number of missing cycles is by measuring the distance between the center of the medullary region and the first visible growth cycle and then dividing by the largest distance between two observed growth cycles (Griebeler, Klein & Sander, 2013; Klein & Sander, 2007; Klein & Griebeler, 2016).

In the current study, applying the latter method might provide a significant error, as the thickness of the growth marks varies distinctly along the sections (Table 2). The third possibility to determine the individual age of the studied bones is thus the estimation of the relative number of the growth cycles based on the comparison between closely related metoposaurids, namely *Dutuitosaurus ouazzoui* from Morocco and *Metoposaurus krasiejowensis* from Poland, which are both well sampled histologically (Steyer et al., 2004; Konietzko-Meier & Klein, 2013; Konietzko-Meier & Sander, 2013; Teschner, Sander & Konietzko-Meier, 2018). Steyer et al. (2004) calculated an individual age for *D. ouazzoui* by counting LAGs in femora, whereas the count of annual growth cycles (Table 3) for *M. krasiejowensis* has been performed for the femora (Konietzko-Meier & Klein, 2013), for the ulnae (Konietzko-Meier & Sander, 2013) and for the humeri (Teschner, Sander & Konietzko-Meier, 2018). In this method the assumption that developmental plasticity does not occur is necessary, especially if working on a small sample size. Konietzko-Meier & Klein (2013) compared the growth rate of *M. krasiejowensis* and *D. ouazzoui* femora and showed that both taxa had a similar growth rate and the correlation between histological framework and bone length is always constant: the longer the bone is the higher is, the number of cycles.

Application of the superimposition method was possible for the humeri of *P. maleriensis*. Therefore, the midshaft section of the smaller specimen (ISIA 73; 65 mm in length) was fitted inside the medullary region of the larger specimen (ISIA 70; 120 mm in length). The number of visible cycles in the smaller bone is one and a half and in the larger humerus it is three. Therefore, the estimation of the relative age, after the addition of the eroded cycle, is at least one and a half for the smaller specimen, and four for the larger specimen. Thus, based on the histological framework, the humerus ISIA 73 likely represents a late juvenile. The ontogenetic stage of the larger specimen (ISIA 70) based on the advanced remodeling process and expectation of achieving the sexual maturity in the fourth year of life should be determined as adult. While comparing the ISIA 73 specimen to a *M. krasiejowensis* humerus of a similar length (67.1 mm in length, one cycle visible; Teschner, Sander & Konietzko-Meier, 2018), the size and corresponding age are similar in both species. However, the Polish locality does not preserve bones belonging to adult specimens and the largest sampled humerus of *M. krasiejowensis* measures 82.6 mm in length and shows two growth cycles (Teschner, Sander & Konietzko-Meier, 2018). For *P. maleriensis* the humerus length corresponds with the histological age: the shorter the bone the ontogenetically younger the specimen and adequately, the larger the bone the older the specimen. There is no data published about the histology of *D. ouazzoui* humeri.

The femoral section (140 mm in length) is in an advanced remodeling stage and some annual growth cycles might have been resorbed. The retrocalculation method cannot be applied here as we only have one femoral section, however an age estimation can be done based on the visible cycles and comparison with *M. krasiejowensis* and *D. ouazzoui* (Konietzko-Meier & Klein, 2013; Steyer et al., 2004). The four visible growth cycles in the ISIA 83 section of *P. maleriensis* correlate to an age of at least four years, with the second cycle showing the beginning of a slowed-down growth rate. It allows to retrocalculate the amount of missing cycles as one and to estimate the age of the bone at five years. It is in contradiction to the femur of *D. ouazzoui* (Steyer et al., 2004), since a femur (AZA131-1; 148 mm) similar in length to the *P. maleriensis* femur is calculated to have 10 years (Steyer et al., 2004). From the Krasiejów locality the largest sectioned specimen of *M. krasiejowensis* is only 92 mm long (UOPB 00912) with an estimation of three to four cycles (Konietzko-Meier & Klein, 2013). The oldest known specimen (UOBS 02123) from the Polish locality is estimated at five years, but is shorter, having only 84 mm in length (Konietzko-Meier & Klein, 2013). The weak correlation between the age and the size represents developmental plasticity typical for lissamphibians and already known for Early Permian stem lissamphibian *Doleserpeton annectens* (Gee, Haridy & Reisz, 2020). This is in contradiction with the conclusion from Konietzko-Meier & Klein (2013), where the developmental plasticity was excluded. However, in the Krasiejów locality, where only long bones of (late) juvenile individuals occur, it is possible that later in ontogeny the growth rate would start to vary, which would result with a disproportion between individual size and age.

The specimen ISIA 200 represents a fragmentary ulna which preserves a partial midshaft and the posterior head of 32 mm and was estimated to 64 mm of total bone length. The cortex is complete, although some cycles might become resorbed. Based on the number of two growth cycles visible, the minimal age corresponds with two years. A sectioned ulna of *M. krasiejowensis* with a total length of 36.9 mm shows two visible cycles (Konietzko-Meier & Sander, 2013). We estimate the minimal age at three years for the *P. maleriensis* ulna.

The ISIA 98 tibia (62 mm in length) shows three growth cycles, which correspond to an age of at least three years. The largest described tibia in *Konietzko-Meier & Klein (2013)* measures only 55 mm in length, however no count of the visible growth marks was published. In the section of *M. krasiejowensis*, two growth cycles are visible (E Teschner, 2020, personal observation), corresponding to a minimum age of two. For the section of *P. maleriensis* we estimated a minimal age of four years for the tibia. In the sampled ulna (ISIA 200) and tibia (ISIA 98) the sections show a large perimedullary region and secondarily widened vascular canals. Based on the amount of the counted growth cycles, both specimens would represent sub-adult individuals.

From the sampled rib specimen (ISIA 199) only a small and fragmentary bone was preserved, thus it is not possible to reconstruct the position of the bone in the skeleton or even the entire rib length. The section preserves three cycles, which indicate a minimum age of three years. The only paper studying the rib histology of *M. krasiejowensis* was published by Gądek (2012), however in this article no age estimation was given. After a personal evaluation (E Teschner, 2020, personal observation) of the rib sections used in Gądek (2012), based on four visible growth cycles a relative age would correspond to four years. While comparing both rib samples, the Indian specimen is ontogenetically of the same age as the Polish individual although *M. krasiejowensis* has one growth cycle more but in *P. maleriensis* the remodeling process is more advanced. It is known from dinosaurs that ribs are a very useful skeletal element for age estimation due to their relatively compact structure and slow growth (Waskow & Sander, 2014; Waskow & Mateus, 2017). On the other hand, it is important to note, that the position of the rib in the skeleton and the sectioning plane are crucial as they influence the amount of visible cycles (Waskow & Mateus, 2017). Therefore, the relative age estimation of the *P. maleriensis* rib is strongly doubtful. To test the expression of the growth marks and interskeletal variability among temnospondyl ribs, and especially Metoposauridae, more studies are necessary.

The ISIA 87 ilium shows no zones and annuli that could be counted. However, as the degree of resorption is very large, it could be assumed that this specimen was not at a juvenile stage anymore. No ilium has been sectioned for comparison from *M. krasiejowensis* or *D. ouazzoui*. The ilium section (ISIA 97) reveals extensive bone remodeling. We assume, that the more metaplastic-like the bone becomes, the older the specimen is. Due to its highly remodeled tissue (Fig. 3D) the ilium may represent an adult individual.

The intercentrum ISIA 198 of *P. maleriensis* has a comparable structure to the intercentrum of *M. krasiejowensis* regarding the large endochondral part, preservation of calcified cartilage in all ontogenetic stages and the periosteal bone being highly vascularized (Konietzko-Meier, Bodzioch & Sander, 2012). Konietzko-Meier, Bodzioch & Sander (2012) studied the histological variability among the intercentra of *M. krasiejowensis*, and created histological ontogenetic stages (HOS). The HOS are based on the characters of the periosteal bone in the vertebra: HOS 1 lacks periosteal ossification and shows no cortex, HOS 2 consists mostly of periosteal bone with an increasing vascularization, HOS 3 is characterized by a decrease in vascularization in the external cortex and HOS 4 shows LAGs in the external cortex (Konietzko-Meier, Bodzioch & Sander, 2012). The studied intercentrum of *P. maleriensis* was growing rapidly and corresponds to the late juvenile/sub-adult HOS 2-3 in comparison to *M. krasiejowensis* (Konietzko-Meier, Bodzioch & Sander, 2012). The only difference is that the *M. krasiejowensis* sections are less diagenetically altered. The calcified cartilage within the trabecular bone represents an intermediate state in the endochondral bone formation, and may indicate incomplete growth and bone immaturity (Hunziker, 1994; Cancedda, Cancedda & Castagnola, 1995; Erlenbacher et al., 1995; Bianco et al., 1998), suggesting the juvenile age of the sampled *P. maleriensis* specimen. However, in Stereospondyli the calcified cartilage in the vertebrae is generally preserved for a long time in the ontogenetic record (Konietzko-Meier, Danto & Gądek, 2014), which also seems to be the case in other vertebrate groups (squamates: Houssaye et al., 2010; sauropterygians: Klein, Canoville & Houssaye, 2019). Therefore, this is not a reliable character that can be used for an age estimation.

The Indian locality shows a wide spectrum of ontogenetic stages with a sample size based on the histological characters with the age range from one and a half to five years, with the only juvenile specimen represented by the small humerus. It is important to note that since we studied disarticulated bones originating most probably from multiple individuals, the individual growth rate of the single skeletal elements cannot be estimated here without sampling bones originating from one individual.

### Climatic and environmental signal in the bones of *Panthasaurus maleriensis*

The climatic signal in osteohistology is the best represented by the growth pattern. Even though every taxon has a genetically fixed growth strategy, the climate can influence the growth pattern. The Late Triassic climate was close to todays’ monsoonal climate (Dubiel et al., 1991; Dickins, 1993; Parrish, 1993). Therefore, the appearance of the zones and annuli in a section most likely represents an alternation of wet and dry periods in an annual cycle. This has been observed in the studied samples, as all limb bones of *P. maleriensis* show an alternation of zones and annuli. The growth in *M. krasiejowensis* is similar to *P. maleriensis*, as both taxa show an alternation of zones and annuli in the cortex. Also, in both taxa no classical LAGs are present, and a slow-down growth rate is represented only by the preservation of numerous resting lines in the annuli. In the juvenile *M. krasiejowensis* specimen, the zones are almost of the same thickness as the annuli (Konietzko-Meier & Klein, 2013; Teschner, Sander & Konietzko-Meier, 2018). In *P. maleriensis* in the inner cortex the annuli are rather thin compared to the zones, and only after achieving the sexual maturity become thicker than zones towards the cortical surface (Table 2). In both juvenile humeri (*M. krasiejowensis* vs. *P. maleriensis*) the size and corresponding age are similar. That confirms the conclusion about the lack of developmental plasticity provided by Konietzko-Meier & Klein (2013) based on the femoral histology observed in individuals from Poland and Morocco. For all three metoposaurid taxa the growth early in ontogeny is relatively uniform, and only after the third growth cycle the growth strategy starts to vary, with *P. maleriensis* slowing the growth rate and achieving sexual maturity and *M. krasiejowensis* continuing the juvenile pattern. In contrast to that are the sectioned femora of *D. ouazzoui* which show an alternation of thick zones and thin annuli and, most importantly, they show regularly deposited LAGs (Steyer et al., 2004). The different growth strategies in the femora of two different genera but of a similar length (about 148 mm) represent different ages: for *Dutuitosaurus* 10 years and for *P. maleriensis* only five years (femora of *Metoposaurus krasiejowensis* are not known in that size).

The different age of achieving sexual maturity for taxa from India and Morocco, could be on the one hand determined by genetic preconditions, however on the other hand, it is known for Lissamphibia that the age of achieving sexual maturity is highly plastic and related to environmental conditions (Miaud et al., 2001). Again, for the Polish taxon the age of sexual maturity is not known, but it was most likely later than in the Indian taxon.

Finally, it can be concluded, that the Polish species *M. krasiejowensis* and the Indian *P. maleriensis* show similar growth pattern, modified by developmental plasticity in later phases of ontogeny. However, common characters could still be observed between the two taxa, as evidenced by the alternation of zones and annuli and the lack of LAGs. Multiple resting lines indicate a partial stagnation of growth. Based on the histological study we assume that the climate was rather mild and not too dry in India during the Late Triassic, which is reflected in the osteohistology during the uninterrupted growth early in the ontogeny. This matches with the geological evidence in the Maleri Formation (Dasgupta, Ghosh & Gierlowski-Kordesch, 2017). Moreover, this would indicate harsher climatic conditions for the Moroccan metoposaurid *D. ouazzoui*, since it shows multiple LAGs in the femora (Dutuit, 1976; Steyer et al., 2004), and a milder climate in India and Poland.

## Conclusion

The material used in this study represents a sufficient sample size including different ontogenetic stages, which allows us to draw conclusions on the growth pattern of *Panthasaurus maleriensis*. All bones consist of lamellar-zonal bone tissue and a cancellous medullary region traversed by secondary trabeculae. The border between the perimedullary region and the cortex is indistinct. A variable degree of collagen-fibers organization in the matrix has been observed resulting in an alternation of highly organized and low vascularized and lower organized and highly vascularized parallel-fibered bone. The cross-sections of the long bones always start with an innermost thick zone with a decreasing thickness of the following zones towards the cortical surface, in contrast to the annuli which increase in size toward the cortical surface and even become thicker than the zones. No clear LAGs can be observed in *P. maleriensis*. However, the annuli deposited in the outer cortex, which do not correspond to the same age, show multiple resting lines. The presence of those resting lines most likely indicates that the individual stayed active during the unfavorable season but temporarily had to cease its growth rate for exogenous reasons such as lack of nutrition or drying out of the water reservoir needed to survive. On the basis of skeletochronology, a strong developmental plasticity can be most likely excluded for *P. maleriensis*. We thus conclude that the presence of the multiple resting lines in the annuli and the absence of LAGs is linked to the environmental and/or climatic influence. Therefore, the Indian *P. maleriensis* would have lived most likely under similar climatic and environmental conditions as the Polish species *M. krasiejowensis* with rather mild climatic conditions making complete cessation of growth rate as indicated by LAGs not necessary. This contrasts with the Moroccan metoposaurid *D. ouazzoui*, where LAGs are occurring even in fast-growing juveniles, and therefore we suggest that the climate during the Late Triassic in Morocco was harsher.

##  Supplemental Information

10.7717/peerj.9868/supp-1 Supplemental Information 1 Accession numbers and specimen localization All listed specimens are stored at the Geological Studies Unit of the Indian Statistical Institute (ISA) in Kolkata, India.

## Additional Information and Declarations

### Data Availability

The following information was supplied regarding data availability: All listed specimens are stored at the Geological Studies Unit of the Indian Statistical Institute in Kolkata, India: ISIA 73 –humerus ISIA 70 –humerus ISIA 83 –femur ISIA 200 –ulna ISIA 98 –tibia ISIA 199 –rib ISIA 87 –ilium ISIA 198 –intercentrum
