# Supplementary material for: Climatic influence on the growth pattern of Panthasaurus maleriensis from the Late Triassic of India deduced from paleohistology
Source: PeerJ. 2020 Sep 8;8:e9868. doi: 10.7717/peerj.9868 (PMC7485487; doi:10.7717/peerj.9868)
Supplement: Supplemental Information 1 — All listed specimens are stored at the Geological Studies Unit of the Indian Statistical Institute (ISA) in Kolkata, India. [file peerj-08-9868-s001.docx]

All listed specimens are stored at the Geological Studies Unit of the Indian Statistical Institute in Kolkata, India. The postal address is as follows: 203 Barrackpore Trunk Road, Kolkata 700108 West Bengal, India; the accession numbers are as following:

ISIA 73 – humerus

ISIA 70 – humerus

ISIA 83 – femur

ISIA 200 – ulna

ISIA 98 – tibia

ISIA 199 – rib

ISIA 87 – ilium

ISIA 198 – vertebra
